# m^6^A Regulates the Stability of Cellular Transcripts Required for Efficient KSHV Lytic Replication

**DOI:** 10.3390/v15061381

**Published:** 2023-06-16

**Authors:** Oliver Manners, Belinda Baquero-Perez, Timothy J. Mottram, Ivaylo D. Yonchev, Christopher J. Trevelyan, Katherine L. Harper, Sarah Menezes, Molly R. Patterson, Andrew Macdonald, Stuart A. Wilson, Julie L. Aspden, Adrian Whitehouse

**Affiliations:** 1 School of Molecular and Cellular Biology, Faculty of Biological Sciences and Astbury Centre of Structural Molecular Biology, University of Leeds, Leeds LS2 9JT, UK; 2 Molecular Virology Unit, Department of Medicine and Life Sciences, Universitat Pompeu Fabra, 08003 Barcelona, Spain; 3 Sheffield Institute for Nucleic Acids, School of Biosciences, University of Sheffield, Firth Court, Western Bank, Sheffield S10 2TN, UK; 4 LeedsOmics, University of Leeds, Leeds LS2 9JT, UK; 5 Department of Biochemistry and Microbiology, Rhodes University, Grahamstown 6140, South Africa

**Keywords:** KSHV, m^6^A methylation, RNA modification, lytic replication, GPCR5A, cell signalling

## Abstract

The epitranscriptomic modification *N*^6^-methyladenosine (m^6^A) is a ubiquitous feature of the mammalian transcriptome. It modulates mRNA fate and dynamics to exert regulatory control over numerous cellular processes and disease pathways, including viral infection. Kaposi’s sarcoma-associated herpesvirus (KSHV) reactivation from the latent phase leads to the redistribution of m^6^A topology upon both viral and cellular mRNAs within infected cells. Here we investigate the role of m^6^A in cellular transcripts upregulated during KSHV lytic replication. Our results show that m^6^A is crucial for the stability of the *GPRC5A* mRNA, whose expression is induced by the KSHV latent–lytic switch master regulator, the replication and transcription activator (RTA) protein. Moreover, we demonstrate that GPRC5A is essential for efficient KSHV lytic replication by directly regulating NFκB signalling. Overall, this work highlights the central importance of m^6^A in modulating cellular gene expression to influence viral infection.

## 1. Introduction

Cellular infection by viruses, including those in the *Herpesviridae* family, is greatly influenced by post-transcriptional regulatory mechanisms that modulate gene expression. Kaposi’s sarcoma-associated herpesvirus (KSHV) is the aetiological agent of Kaposi’s sarcoma and two further lymphoproliferative disorders, primary effusion lymphoma and multicentric Castleman’s disease [1,2,3,4]. KSHV undergoes a complex biphasic life cycle encompassing latent persistence and lytic replication phases. During latency, the virus enters a state of transcriptional dormancy, where expression is limited to a few latent genes [5,6,7]. Certain factors, including stress and immunosuppression, trigger the reactivation of the virus into the lytic phase, where over 80 viral genes are expressed, leading to the production of infectious virions [8,9]. The replication and transcription activator (RTA) protein, expressed by the open reading frame ORF50, is essential and sufficient for the process of reactivation, and serves as a molecular switch from latent to lytic replication phases [10,11,12]. Notably, both the latent and lytic replication phases are essential for KSHV-mediated tumorigenicity [13,14,15].

Amassing evidence suggests that viral life cycles are greatly influenced by a group of chemical modifications of RNA, collectively known as the epitranscriptome [16]. The most common epitranscriptomic modification of mRNA is *N*^6^-methyladenosine (m^6^A), whose dynamics are controlled by specific cellular machinery that install, remove and decode the modification [17,18,19,20]. The multicomponent m^6^A writer complex comprises the catalytically active subunits, methyltransferase-like 3 (METTL3) and methyltransferase-like 4 (METTL14), which methylate the central adenosine residue at the consensus DRACH sequence (D = A/G/U, R = A/G and H = A/C/U) [12]. Additional components, such as WTAP, RBM15, VIRMA and ZC3H13, are responsible for selectivity, localisation and structural integrity [21,22,23]. Conversely, two RNA demethylases known as m^6^A erasers, α-ketoglutarate-dependent dioxygenase alkB homolog 5 (ALKBH5) [24] and fat mass obesity protein (FTO) [25], revert m^6^A back to adenosine residues. Although the impact and extent of demethylation carried out by the m^6^A ‘erasers’ ALKBH5 and FTO is debated, it is widely accepted that at least some m^6^A residues can be reversed back to adenosine [26]. Importantly, the dynamic reversible addition and removal of m^6^A allows rapid adjustment of mRNA fate, and thus regulatory control. Finally, m^6^A exerts its influence over mRNAs in *cis* by recruiting RNA binding proteins, known as m^6^A readers, which in turn modulate the structure, splicing, nuclear export, stability and translation of the transcript [27]. The most widely characterised group of m^6^A readers, the YT521-B homology (YTH)-domain-containing proteins, directly bind m^6^A in target RNAs through an aromatic cage [28]. In contrast, a second group of m^6^A readers, including several hnRNPs, preferentially bind m^6^A-modified RNAs through an m^6^A switch mechanism, an event in which m^6^A modification remodels the local RNA structure [29]. A myriad of m^6^A readers may therefore exist that enable widespread regulatory control over gene expression [19], affecting many biological pathways. Importantly, dysregulation of these pathways is now implicated in a wide variety of disease states, including viral infection.

The ability of m^6^A to dynamically regulate gene expression offers unique possibilities for viruses to modulate viral and host gene expression, and also for the host to regulate a response to infection [30]. Recent studies demonstrate that m^6^A is deposited upon the RNAs transcribed by a diverse range of both RNA and DNA viruses [31,32,33,34], and highlight distinct pro- and antiviral roles, indicating widespread regulatory control over viral life cycles [16,34]. For example, recent work has shown that Epstein-Barr virus infection targets the host m^6^A pathway to attenuate the innate immune response and also promote cell survival [35,36]. Similarly, KSHV lytic replication induces vast changes in m^6^A distribution, with important consequences for both host and viral gene expression [32,37,38,39]. However, whilst the redistribution of m^6^A topology on cellular transcripts in response to viral infection has been reported, and groups of cellular RNAs have been characterized by gene ontology studies, very few studies have assessed the impact of individual differentially modified m^6^A sites within specific cellular transcripts during viral infection. Therefore, the aim of this study was to identify and assess the impact of individual m^6^A residues in cellular mRNAs during KSHV reactivation, to help towards a clear understanding of the role of viral epitranscriptomics.

In this study, we investigated the impact of specific m^6^A residues in host transcripts upon KSHV reactivation, by identifying transcripts with altered methylation profiles between latent and lytic replication programmes. We identified a subset of cellular mRNAs that heavily increase in both m^6^A content and abundance during KSHV lytic replication. Functional interrogation suggests that these genes may encode key cellular factors involved in enhancing lytic replication, and their expression can be reduced through the disruption of m^6^A sites in these transcripts, indicating that these m^6^A sites have potential for therapeutic targeting.

## 2. Materials and Methods

### 2.1. Mammalian Cell Culture

HEK 293T cells were obtained from the ATCC and were maintained in Dulbecco’s modified Eagle’s medium (DMEM) containing 10% foetal bovine serum (FBS) and 1% penicillin/streptomycin [40]. TREX BCBL1-Rta cells, a KSHV-infected, primary effusion lymphoma B lymphocyte cell line engineered to express C-myc-RTA under a doxycycline inducible promoter, was kindly provided by Dr JU Jung, University of Southern California [41]. TREX-BCBL1-Rta cells were cultured in RPMI 1640 medium (Sigma, St. Louis, MO, USA) with glutamine (Gibco, Paisley, UK) supplemented with 10% FBS, 1% P/S (Gibco Paisley, UK) and 100 μg/mL hygromycin B (ThermoFisher, Waltham, MA, USA). TREX-BCBL1-Rta cells transduced with lentivirus were cultured in medium further supplemented with 3 μg/mL puromycin (ThermoFisher, Waltham, MA, USA). KSHV lytic reactivation was induced in TREX-BCBL1-Rta cells using 2 μg/mL doxycycline hyclate (Sigma, St. Louis, MO, USA). Cell lines were maintained at 37 °C in a 5% CO_2_ atmosphere. Plasmids were transfected with Lipofectamine 2000 (ThermoFisher, Waltham, MA, USA) in a 1:2 ratio in Opti-MEM media (Gibco, Paisley, UK), as previously described [42].

### 2.2. Antibodies and Plasmids

Antibodies used for immunoblotting: C-Myc (Sigma M4439 1:1000), FLOT1 (CST D2V7J 1:1000, Leiden, Netherlands), FTO (Abcam ab126605 1:5000, Cambridge UK), GAPDH (Proteintech 60004-1-Ig 1:5000, Manchester, UK), GFP (Living Colours 632381 1:1000, Kusatsu, Japan), GPRC5A (Atlas HPA007928 1:500, Broma, Sweden), NFκB (CST D14E12, Leiden, Netherlands), phospho-NFκB (CST 93H1, Leiden, Netherlands), ORF57 (Santa Cruz sc-135747 1:1000, Dallas, TX, USA), ORF65 (CRB crb2005224 1:100, Cambridge, UK), STC1 (Proteintech 20621-1-AP 1:500, Manchester, UK)), WTAP (Abcam ab195380 Rabbit 1:1000, Cambridge, UK). pVSV.G and psPAX2 were gifted by Dr Edwin Chen (University of Westminster, London, UK). GFP, GFP-ORF50 and GFP-ORF57 plasmids are previously described [43,44]. pRL-TK (Addgene #E2241) and pNFkB-ConA plasmids were gifted by the Macdonald group, previously described in [45]. The pLENTI-CMV-GFP-PURO construct was purchased from Addgene (#17448). GPRC5A-, GPRC5A-FLAG- and GPRC5A-expressing plasmids were generated by PCR of the entire coding sequence of GPRC5A from TREX-BCBL-1 cDNA and cloned using the NEBuilder HIFI DNA assembly kit (NEB, Hitchin, UK) into pLENTI-CMV-GFP-PURO. PLKO.1 TRC cloning vectors expressing shRNA sequences were purchased from Merck or Dharmacon: FTO (TRCN0000246247), GPRC5A KD1 (TRCN0000005628), GPRC5A KD2 (TRCN0000005632), WTAP (TRCN0000231423), YTHDF1 Sigma (TRCN0000286871). pLENTI-CMV-GPRC5A was mutated to remove m6A sites (A57G, G120T, C174T, A264G) using the Q5 site-directed mutagenesis kit (NEB, Hitchin, UK), according to the manufacturer’s instructions. Four consecutive rounds of site-directed mutagenesis were performed to mutate each of the individual bases. Primers are listed in Table S1.

### 2.3. RNA Extraction, cDNA Synthesis and qPCR

Total RNA was isolated from cells using the Monarch Total RNA Miniprep kit (NEB, Hitchin, UK) according to the manufacturer’s protocol. Subsequently, 1 µg RNA was reverse transcribed to cDNA using the LunaScript RT SuperMix kit (NEB). qPCR was performed in 20 µL reactions containing diluted cDNA, GoTaq qPCR MasterMix (Promega, Southampton, UK) and the desired primer set. qPCR data were acquired using Rotorgene Q 6000 1.7 software and analysed using the ΔΔCT method relative to GAPDH, as previously described [46].

### 2.4. Immunoblotting

Protein samples were separated on 10–15% polyacrylamide gels and transferred to Amersham nitrocellulose membranes (GE healthcare, Hatfield, UK) by Trans-blot Turbo Transfer system (Bio-Rad, Watford, UK). Membranes were blocked using TBS + 0.1% Tween 20 with 5% (*w*/*v*) skimmed milk powder, and probed with desired primary antibodies and secondary horseradish-peroxidase-conjugated IgG antibodies at 1:5000 (Dako Agilent, Santa Clara, CA, USA). Protein bands were detected using a G:BOX (Syngene) after treating membranes with ECL Western blotting substrates (Promega). Densitometry was performed using ImageJ 1.51 software.

### 2.5. Immunofluorescence

TREx-BCBL1-Rta cells were seeded onto coverslips treated with poly-l-lysine (Sigma, St. Louis, MO, USA), fixed for 15 min in 4% paraformaldehyde and permeabilised with PBS + 1% Triton-X-100, as previously described [47]. Subsequent incubations were carried out at 37 °C for 1 h in a humidified chamber. Cells were blocked using PBS with 1% BSA prior to incubation with the desired primary antibodies and then Alexa Fluor-conjugated secondary antibodies. (Invitrogen 1:500, Paisley, UK). Coverslips were mounted onto slides using Vectashield Hardset Mounting Medium with DAPI (Vector laboratories, Newark, NJ, USA). Images were acquired using a Zeiss LSM880 inverted confocal microscope and analysed using ZEN 2009 imaging software (Carl Zeiss, Jena, Germany).

### 2.6. Luciferase Assays

HEK 293T cells were seeded for 24 h before transfection with pNFkB-ConA (containing tandem kB-response element repeats) and pRL-TK Renilla transfection control luciferase reporter plasmids. After 24 h, samples were lysed with 1× Passive Lysis Buffer (Promega, Southampton, UK) and relative luciferase activity measured using Dual Luciferase Stop and Glo reagents (Promega, Southampton, UK), as directed by the manufacturer.

### 2.7. Viral Reinfection Assays

Virus-containing supernatant was harvested from TREx-BCBL1-Rta cells reactivated for 72 h and used to infect naïve HEK 293T cells in DMEM at a 1:1 ratio. At 24 h post addition of viral supernatant, cells were collected, and total RNA was isolated and reverse transcribed into cDNA for qPCR analysis of ORF57 mRNA levels.

### 2.8. Lentiviral Transduction

Lentiviruses were produced by co-transfection of HEK 293T cells with 1.2 μg of lentiviral vector and 0.65 μg of packaging plasmids psPAX2 and VSV.G. After 72 h, supernatant-containing lentivirus was filter-sterilised and combined with TREx-BCBL1-Rta cells in 8 μg/mL polybrene (Merck Millipore, Burlington, MA, USA). After 8 h, the cells were placed in fresh medium. At 48 h post transduction, cells were placed under selection with medium containing 3 μg/mL puromycin (Gibco, Paisley, UK), which was replaced every 2–3 days, as previously described [48]. To confirm that depletion by lentivirus transduction had no effect on cell viability and proliferation, TREx BCBL1-Rta cells stably expressing scrambled or targeted shRNAs were seeded into a 6-well plate at 0.2 × 10^6^ cells/well with 2 mL fresh RPMI selection medium. Cells were then grown for 48 h and counted at each 24 h interval.

### 2.9. m^6^A Immunoprecipitation

m^6^A IPs were carried out as described previously [32]. Briefly, TREx-BCBL1-Rta cell total RNA was fragmented into 100–200 nucleotide segments using RNA fragmentation reagents (Ambion, Austin, TX, USA) and sodium-acetate-precipitated overnight at −80 °C. A 5% input was collected prior to immunoprecipitation. The remaining RNA was combined with 25 μL of Magna ChIP Protein A+G magnetic beads (Merck Millipore, Burlington, MA, USA) coated in 5 μL of anti-m6A antibody (Merck Millipore, Burlington, MA, USA) and incubated at 4 °C overnight with rotation. RNA from inputs and m^6^A immunoprecipitations was eluted using proteinase K (ThermoFisher, Waltham, MA, USA) followed by Trizol LS: chloroform extraction. RNA immunoprecipitations and inputs were converted to cDNA using the LunaScript RT SuperMix kit (NEB, Hitchin, UK). m^6^A-immunoprecipitated samples were normalised to their respective input samples, and m^6^A content at a particular region was calculated relative to an unmodified control region within the same transcript.

### 2.10. RNA Stability Assays

HEK 293T were transfected with appropriate plasmids for 48 h, then treated with 2.5 μg/mL of actinomycin D (ThermoFisher, Waltham, MA, USA) and samples collected at the desired time points. Total RNA was isolated prior to reverse transcription and qPCR analysis of specific transcripts, as described previously. However, mRNA expression was normalised to 18s rRNA rather than GAPDH, as the rRNA is more stable, as previously described [32].

### 2.11. Immunoprecipitation Assays

GFP- or GPRC5A-GFP-transduced TREx-BCBL1-Rta cells were lysed in GFP-TRAP buffer (10 mM TrisHCl pH 7.5, 150 mM NaCl, 0.5 mM EDTA, 0.5% NP40) and incubated with 25 µL GFP-Trap Agarose beads (Chromotek, Manchester, UK) for 3 h at 4 °C. Beads were washed 3 times in GFP-TRAP buffer and immunoprecipitated proteins were eluted by resuspension in 30 μL 2× Laemmli sample buffer and boiled at 90 °C for 10 min. Finally, the proteins were separated by SDS-PAGE and detected by Western blotting, as described previously [49].

### 2.12. Quantitative Proteomics

Parental TREx-BCBL1-Rta cells and GPRC5A-FLAG-expressing TREx-BCBL1-Rta cells were lysed in immunoprecipitation buffer (150 mM NaCl, 2 mM EDTA, 0.5% Triton-X 100, 0.5 mM DTT, 10 mM Tris pH 7.4 and 1× protease and phosphatase inhibitors). Lysates were centrifuged at 12,000× *g* for 10 min and the supernatant collected. A 5% input was taken at this stage and mixed with 15 μL 2× Laemmli sample buffer for downstream applications. Subsequently, 15 μL Protein A or G Dynabeads (ThermoFisher, Waltham, MA, USA) were pre-prepared by washing 3 times in immunoprecipitation buffer, and then resuspended in 200 μL immunoprecipitation buffer and coated with 2 μL FLAG M2 (Merck Millipore, Burlington, MA, USA) or IgG (Merck Millipore, 12–370, Burlington, YSA) control antibody. Beads were rotated for 1 h at 4 °C, washed 3 times with immunoprecipitation buffer and incubated with lysates overnight at 4 °C. The following day, beads were washed 3 times in immunoprecipitation buffer and analysed at the University of Bristol Proteomics Facility for tandem mass tagging (TMT) coupled with liquid chromatography and mass spectrometry analysis (LC-MS/MS), as previously described [50]. A Microsoft Excel spreadsheet containing the results of a Sequest search against the Uniprot Human Database was generated. Data from two independent replicates were grouped before filtering the best interactors. Common contaminants identified via database were removed and data filtered to satisfy an FDR of less than 5%. Protein abundance ratios were calculated between GPRC5A-FLAG cells and TREx cells for both latently and lytically infected cells. Proteins were filtered by a minimum abundance of 100 in GPRC5A-FLAG cells and a minimum net abundance of 50 after subtraction of background abundances in TREx cells. An abundance ratio of 2 was set for GPRC5A-FLAG cells relative to TREx cells in latency. However, a 1.4-fold enrichment was used for GPRC5A-FLAG cells relative to TREx cells in lytic replication, as fewer proteins were detected due to the action of virus-mediated host cell shutoff. Proteins with enriched GPRC5A interaction in the lytic phase were identified using a minimum 1.5-fold interaction in lytic cells compared to those undergoing latent replication after subtraction of the TREx cell background. The 51 proteins in the latent phase, 51 proteins in the lytic phase and 25 proteins enriched in the lytic phase that met these strict cut-offs were subjected to STRING protein–protein interaction networks functional enrichment analysis.

### 2.13. Statistical Analysis

Unless otherwise stated, graphical data shown represent mean ± standard deviation of mean (SD), using 3 or more biologically independent experiments. Differences between means were analysed by unpaired Student’s t test calculated using the Graphpad Prism 9 calculator. Statistics are considered significant at *p* < 0.05, with * *p* < 0.05, ** *p* < 0.01, *** *p* < 0.001.

### 2.14. Data Availability

The mass spectrometry proteomics data have been deposited into the ProteomeXchange Consortium via the PRIDE partner repository. TREx-BCBL1-RTA GPRC5A-FLAG Immunoprecipitation LC-MS. Project accession: PXD039669. TREx-BCBL-Rta RNA-seq, 2 replicates at KSHV infection time points of 0 h (accessions GSM2460361, GSM2754227) and 48 h (accessions GSM2460363, GSM2754229) were used in the study.

## 3. Results

### 3.1. Differential m^6^A Status of Host Cell Transcripts Corresponds to Expression Levels during KSHV Lytic Replication

m^6^A-seq analysis obtained from KSHV-infected TREx-BCBL1-Rta cells, a latently infected KSHV B-lymphocyte cell line that expresses a Myc-tagged viral RTA under the control of a doxycycline-inducible promoter, identified large-scale remodelling of the host cell m^6^A epitranscriptome during reactivation of the KSHV lytic replication cycle [32]. However, given that many of these changes in m^6^A peaks were concordant with changes in mRNA expression, we specifically searched for mRNAs with differentially modified m^6^A sites relative to an m^6^A peak in the same transcript whose methylation status remained stable. Notably, we identified 58 transcripts that contained both stable and differential m^6^A peaks, which we prioritised (Table S2); examples of differential m^6^A modification within specific transcripts during latent and lytic replication phases are demonstrated in Figure S1A–D. Ingenuity pathway analysis (IPA) was then performed to assess whether these differentially m^6^A-modified mRNAs were associated with distinct functional networks and cellular activities (Figure S2). The strongest predicted canonical pathways involved RNA processing and cell signalling. Similarly, predicted molecular and cellular functions and diseases identified cell-to-cell signalling, gene expression and cancer-related pathways.

**Figure 1:**
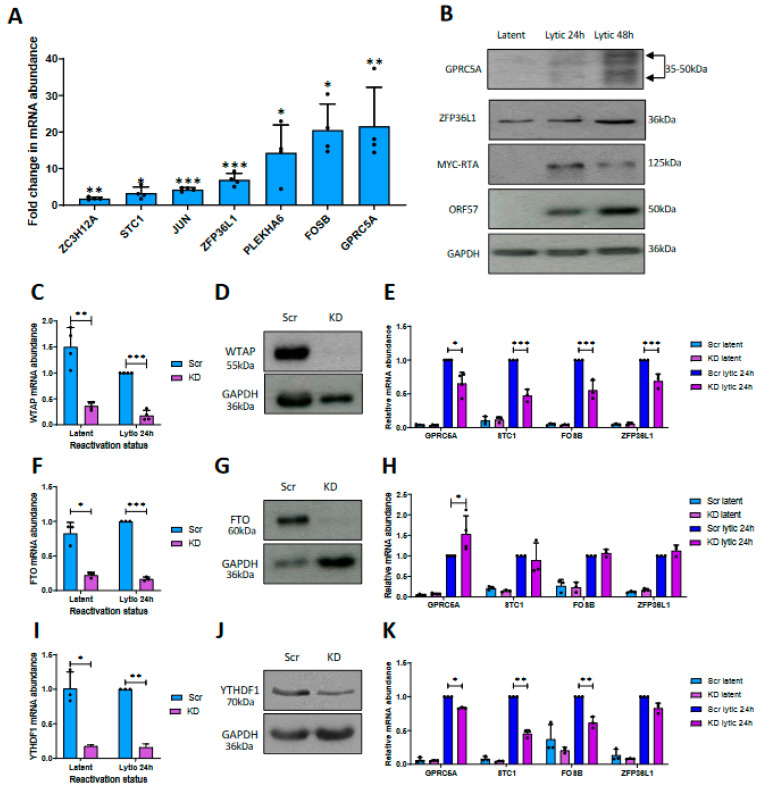
m^6^A affects the abundance of host transcripts upregulated during KSHV reactivation. (**A**) RT-qPCR analysis of cellular m6A-modified transcripts in TREX-BCBL1-Rta cells induced for 24 h compared to latent levels. (**B**) Representative Western blot of ZFP36L1 and GPRC5A protein levels in latent and reactivated TREX-BCBL1-Rta cells. (**C**) RT-qPCR analysis of WTAP RNA levels in latent and lytic WTAP shRNA-treated TREX-BCBL1-Rta cells. (**D**) Representative Western blot of WTAP protein levels in Scr- and WTAP-shRNA-treated latent TREX-BCBL1-Rta cells. (**E**) RNA levels of GPRC5A, STC1, FOSB and ZFP36L1 in Scr- or WTAP shRNA-treated latent and induced TREX-BCBL-1 cells. (**F**) RT-qPCR analysis of FTO RNA levels in latent and lytic FTO shRNA-treated TREX-BCBL1-Rta cells. (**G**) Representative Western blot of FTO protein levels in Scr- and FTO-shRNA-treated latent TREX-BCBL1-Rta cells. (**H**) RNA levels of GPRC5A, STC1, FOSB and ZFP36L1 in Scr- or FTO shRNA-treated latent and induced TREX-BCBL1-Rta cells. (**I**) RT-qPCR analysis of YTHDF1 RNA levels in latent and lytic YTHDF1 shRNA-treated TREX-BCBL1-Rta cells. (**J**) Representative Western blot of YTHDF1 protein levels in Scr- and YTHDF1-shRNA-treated latent TREX-BCBL1-Rta cells. (**K**) RNA levels of GPRC5A, STC1, FOSB and ZFP36L1 in Scr- or YTHDF1 shRNA-treated latent and induced TREx-BCBL1-Rta cells. In (**A**,**C**,**E**,**F**,**H**,**I**,**K**), data are presented as mean ± SD. * *p* < 0.05, ** *p* < 0.01, *** *p* < 0.001. All repeats are biological (n = 3).

We then prioritised cellular mRNAs that increased in their expression during lytic replication, as these contrast with the majority of cellular transcripts, which are degraded by the viral SOX endonuclease during KSHV-mediated host cell shutoff [51,52]. To confirm an increase in expression of these host mRNAs during lytic replication, we selected a number of transcripts with the greatest changes in m^6^A content, and confirmed their expression levels by RT-qPCR, comparing levels during latency and 24 h post induction of lytic replication (Figure 1A). All mRNAs selected for investigation were significantly upregulated in the lytic phase compared with latency; most notably, *GPRC5A* and *FOSB* transcripts were 19-fold more abundant in the lytic phase. Immunoblotting of cell lysates from latent and lytic TREx-BCBL1-Rta cells using GPRC5A- and ZFP36L1-specific antibodies confirmed that the changes in mRNA expression led to a similar increase in protein levels (Figure 1B). These changes in expression are consistent with previous studies and in other KSHV-infected cell lines. Figure S1E shows a strong correlation between the DESeq2-calculated log2-fold changes in gene expression within this study and published BCBL1-R KSHV data [38]. In addition, RT-qPCR analysis confirmed an increase in the expression of *GPRC5A* and *FOSB* transcript levels during lytic replication in BCBL-1 and HEK 293 rKSHV.219 cell lines [53] (Figure S1F).

Given a potential link between the increase in cellular transcript abundance and increased m^6^A content during KSHV lytic reactivation (Table S2), we speculate that disruption of m^6^A dynamics, through depletion of the host cell m^6^A machinery components, may alter mRNA levels. To examine whether mRNA abundance and m^6^A levels were functionally linked, TREx-BCBL1-Rta cells were stably transduced with lentivirus-based shRNAs depleting three different components of the host m^6^A machinery involved in loading, removal and recognition of the RNA modification, namely, the m^6^A writer complex component WTAP, the m^6^A eraser FTO or the m^6^A reader YTHDF1, respectively. RT-qPCR and immunoblotting confirmed the reduction in WTAP (Figure 1C,D), FTO (Figure 1F,G) and YTHDF1 (Figure 1I,J) by 75%, 73% and 82% relative to a scrambled control. The results of densitometry analysis of Western blots are shown in Figure S1G. To assess whether disruption of the m^6^A machinery affected the increased abundance of the cellular transcripts, mRNA levels were compared by RT-qPCR in the WTAP-, FTO- and YTHDF1-depleted TREX-BCBL1-Rta cells relative to scrambled controls during latency or lytic replication (Figure 1E,H,K). Notably, WTAP depletion significantly reduced the abundance of the cellular m^6^A-modified mRNAs, i.e., *GPRC5A*, *STC1*, *FOSB* and *ZFP36L1*, suggesting that methylation is important for their abundance during KSHV lytic replication. In contrast, no changes in expression levels were observed for the non-methylated transcript, *HIPK3*, in the WTAP knockdown cell line (Figure S1H). Conversely, the depletion of the m^6^A eraser FTO enhanced the upregulation of *GPRC5A* by ~50% (Figure 1K). However, we did not detect a significant increase in the levels of *STC1*, *FOSB* or *ZFP36L1*. Given that FTO is not a global m^6^A eraser, and does not demethylate all m^6^A sites [54], these data suggest that FTO may only target *GPRC5A* among these four transcripts for demethylation. Finally, as m^6^A provides a signal upon RNAs to be decoded by m^6^A readers, which in turn direct the transcript towards its predetermined biological fate, abrogation of m^6^A ‘writing’ or ‘reading’ should lead to similar mRNA phenotypes. Interestingly, knockdown of the m^6^A reader YTHDF1 recapitulated the effect of WTAP depletion, with diminished levels of *GPRC5A*, *STC1*, *FOSB* and *ZFP36L1* observed. This may reflect dosage-dependent redundancy when all three readers are equivalently co-expressed in the same cell type [55,56]. Finally, proliferation assays were performed to confirm that depletion of WTAP and YTHDF1 had no effect on cell viability or proliferation. The results show that depletion of these m^6^A machinery components had no effect on growth rates of knockdown cell lines compared to a scrambled control over a 48 h period (Figure S1I). Taken together, these data suggest that m^6^A is linked to the abundance and upregulation of four m^6^A-modified cellular mRNAs: *GPRC5A*, *STC1*, *FOSB* and *ZFP36L1*.

### 3.2. m^6^A Sites within GPRC5A mRNA Regulate Its Stability

**Figure 2:**
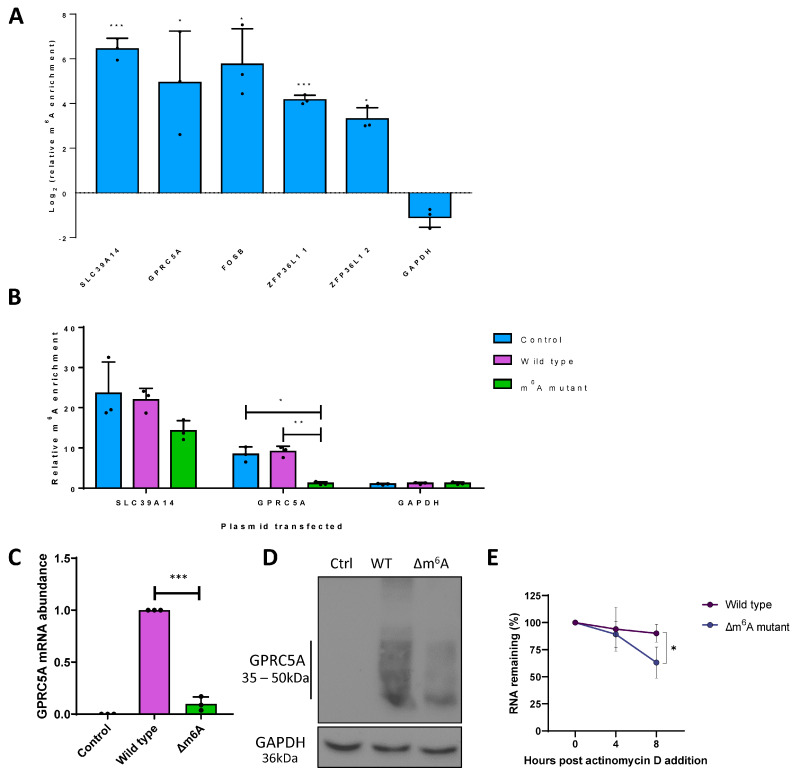
m^6^A sites within the cellular GPRC5A transcript are essential for its stability. (**A**) m^6^A IP-qPCR analysis of cellular transcripts or control RNAs in latent TREX-BCBL1-Rta cells showing log2-fold enrichment of m^6^A-modified region relative to a non-methylated region in *cis*. Two regions were analysed within *ZFP36L1*, as this mRNA contains a wide flat m^6^A peak spanning several hundred bps. (**B**) m^6^A IP-qPCR analysis of *GPRC5A* or control RNAs in GPRC5A wild-type or Δm^6^A mutant-transfected HEK 293T cells showing fold enrichment of the m^6^A-modified region relative to a non-methylated region in cis. (**C**) RT-qPCR analysis of *GPRC5A* RNA in cells transfected with control plasmid, a wild type of GPRC5A-expressing constructs. (**D**) Representative Western blot of GPRC5A protein levels in cells transfected with no plasmid, wild-type or Δm^6^A GPRC5A-expressing constructs. (**E**) RT-qPCR analysis of wild-type or Δm^6^A GPRC5A RNA in cells treated with actinomycin D for 0, 4 or 8 h. In (**A**,**B**,**C**,**E**), data are presented as mean ± SD. * *p* < 0.05, ** *p* < 0.01, *** *p* < 0.001. All repeats are biological (n = 3).

Although depletion of WTAP, FTO and YTHDF1 and the consequent disruption of m^6^A dynamics modulated *GPRC5A*, *STC1*, *FOSB* and *ZFP36L1* transcript levels, it remained unclear whether these changes are direct *cis*-acting effects on these mRNAs resulting from perturbation of the m^6^A machinery. Therefore, we used m^6^A immunoprecipitation coupled with RT-qPCR (m^6^A IP-qPCR) to validate the presence of m^6^A methylation on these cellular transcripts (Figure 2A). The transcripts *SLC39A14* and *GAPDH* were used as positive and negative controls, as the former contains a prominent and well-described m^6^A site, while the latter is known to be an unmodified transcript [18]. For each gene, two primer sets were designed, with one spanning the putative m^6^A peak and the other spanning a region lacking m^6^A according to the m^6^A-seq data. m^6^A peaks were confirmed by enrichment of the m^6^A spanning the null primer sets by m^6^A IP-qPCR, using RNA fragmented to ~200 nt to ensure good spatial resolution. Prominent m^6^A modification was detected in *SLC39A14*, *GPRC5A*, *FOSB* and *ZFP36L1* mRNAs, while no enrichment was identified for *GAPDH*, validating the m^6^A modification of *GPRC5A*, *FOSB* and *ZFP36L1* at the expected locations. Unfortunately, this method could not be used for *STC1*, as no suitable primer sets could be designed that were able to amplify the proposed methylated region efficiently.

GPRC5A belongs to a family of proteins that are often reported to function at the top of the signalling cascade, although their role in virus infection is yet to be fully determined. Therefore, we prioritised GPRC5A for further investigation, as it had a single narrow differentially modified m^6^A peak (Figure S1), and its abundance was altered during lytic replication of TREx-BCBL1-Rta cells depleted for WTAP, FTO or YTHDF1. The four potential DRACH sites within the *GPRC5A* m^6^A peak were altered by site-directed mutagenesis, termed Δm^6^A, with no alteration in amino acid sequence and minimal changes to the DNA sequence (Figure S3). To confirm that mutation of the proposed m^6^A DRACH sites within the single dysregulated m^6^A-seq peak of GPRC5A reduced its m^6^A content, m^6^A IP-qPCR was performed after transfection of wild-type or Δm^6^A GPRC5A-expressing plasmids into HEK 293T cells (Figure 2B). Importantly, both endogenous and wild-type *GPRC5A* mRNA showed clear m^6^A enrichment consistent with previous experiments. However, the mutated *GPRC5A* mRNA showed no enrichment in this region, comparable with the known unmethylated transcript *GAPDH*, highlighting the complete removal of the m^6^A site. Notably, deletion of m^6^A within GPRC5A recapitulated the effects of WTAP or YTHDF1 depletion, as shown previously, as expressions of *GPRC5A* mRNA and protein were significantly attenuated in HEK 293T cells transfected with the Δm^6^A GPRC5A construct compared to the wild-type GPRC5A construct (Figure 2C,D). Given that both GPRC5A protein and mRNA were affected equally by the deletion of m^6^A, we hypothesise that methylation of the *GPRC5A* transcript may be important for its stability. However, it cannot be ruled out that the mutations themselves may be affecting the stability or folding of the transcript directly. To further assess this phenotype, HEK 293T cells were transfected with wild-type or Δm^6^A GPRC5A constructs and treated with the transcriptional inhibitor actinomycin D for 0, 4 or 8 h. RT-qPCR analysis showed a significant reduction in stability in the Δm^6^A mutant, resulting in only 63% of Δm^6^A *GPRC5A* after 8 h of actinomycin D treatment compared with 90% for the wild-type construct (Figure 2E). Taken together, these results suggest that a differentially modified m^6^A peak, containing four potential DRACH sites, within the *GPRC5A* mRNA is important for its abundance by enhancing its stability.

### 3.3. RTA Transactivates the GPRC5A Promoter

**Figure 3:**
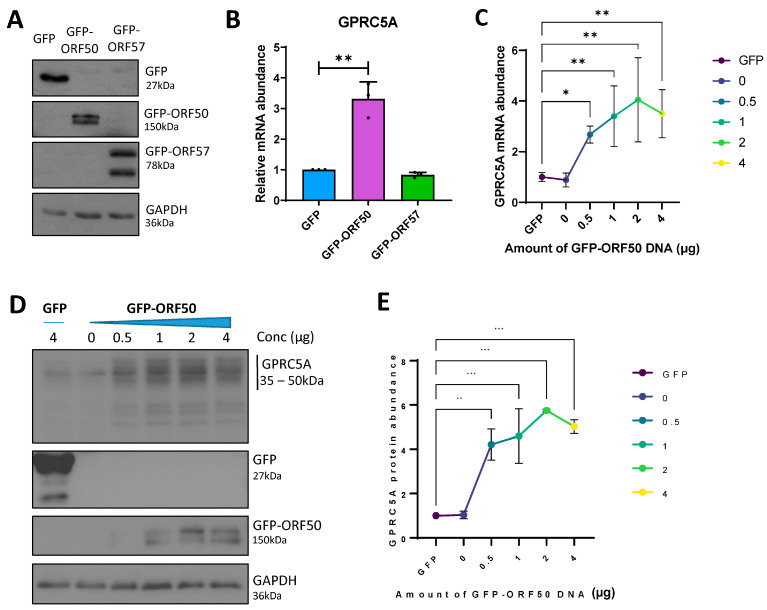
GPRC5A is induced by the KSHV lytic transactivator, RTA. (**A**) Representative Western blot showing expression of transfected GFP-tagged viral proteins ORF50 and ORF57 in naïve uninfected HEK 293T cells. (**B**) RT-qPCR analysis of GPRC5A RNA levels in viral-protein-transfected HEK 293T cells. (**C**) RT-qPCR analysis of GPRC5A RNA levels in cells transfected with increasing concentrations of GFP-ORF50 DNA. (**D**) Representative Western blot of GPRC5A protein levels in cells transfected with a range of GFP-ORF50 DNA concentrations. (**E**) Densitometry quantification of immunoblots was performed using ImageJ software, and is shown as a percentage relative to the loading control, GAPDH. In (**B**,**C**,**E**), data are presented as mean ± SD. * *p* < 0.05, ** *p* < 0.01, *** *p* < 0.001. All repeats are biological (n = 3).

Cellular transcripts that increase in abundance during KSHV lytic replication contrast with the majority of host mRNAs, which are degraded by the KSHV-encoded endonuclease, SOX [51,52]. As a result, upregulated transcripts are more likely to be functionally relevant in the KSHV lytic replication cycle. Two KSHV lytic proteins with the ability to increase the abundance of host transcripts during the early stages of lytic replication are the lytic master regulator RTA, encoded by ORF50, and the mRNA processing factor, ORF57. RTA is a transcription factor able to transactivate both viral and cellular promoters [57], whereas ORF57 can bind and stabilise cellular mRNAs, enhancing their RNA processing and translation [58]. To determine whether the upregulation of the m^6^A-modified *GPRC5A* transcript is dependent on these viral factors, we transfected constructs expressing GFP-tagged-RTA [43] or -ORF57 [44] into uninfected HEK 293T cells, and confirmed their ectopic expression by Western blotting (Figure 3A). Our results show a threefold increase in *GPRC5A* mRNA, but only in the presence of GFP-tagged-RTA expression (Figure 3B). Furthermore, by transfecting increasing amounts of GFP-ORF50 DNA into HEK 293T cells, we identified a dose-dependent increase in *GPRC5A* mRNA levels up to fourfold (Figure 3C). Additionally, the increase in *GPRC5A* mRNA was accompanied by a sixfold increase in GPRC5A protein production (Figure 3D,E). Although not as high as the 18-fold increase in GPRC5A expression in B cells undergoing KSHV lytic replication, we hypothesise that increases in *GPRC5A* m^6^A levels, as well as the higher RTA protein levels attained, may account for these differences. Interestingly, the finding that RTA increases the expression of GPRC5A is supported by previous genome-wide RTA ChIP-seq studies, which identified RTA binding sites within the GPRC5A promoter (Figure S4) [59]. Together, these results suggest that the KSHV protein RTA induces the transcription of *GPRC5A* mRNA, whose post-transcriptional stability is further regulated by m^6^A modification.

### 3.4. GPRC5A Is Required for Efficient KSHV Lytic Reactivation

**Figure 4:**
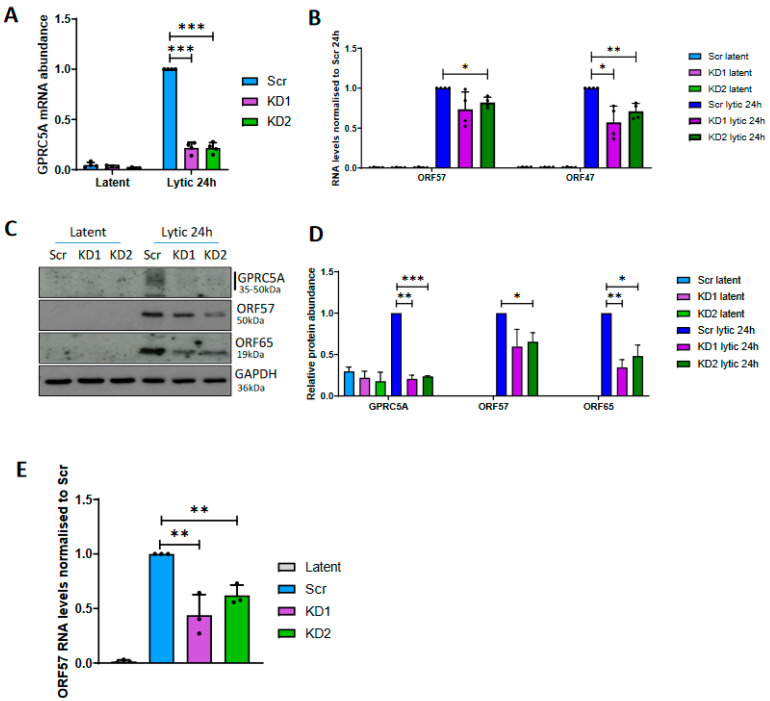
GPRC5A depletion reduces KSHV lytic replication. (**A**) RT-qPCR analysis of GPRC5A RNA levels in latent and lytic GPRC5A shRNA-treated TREX-BCBL1-Rta cells. (**B**) RT-qPCR analysis of viral RNAs *ORF57* and *ORF47* in latent and lytic GPRC5A-depleted cells. (**C**) Representative Western blot of viral proteins ORF57 and ORF65 in latent and lytic GPRC5A-depleted cells. (**D**) Densitometry quantification of immunoblots was performed using ImageJ software, and is shown as a percentage relative to the loading control, GAPDH. (**E**) RT-qPCR of *ORF57* RNA levels in HEK 293T cells treated with virus-containing supernatant harvested from scrambled or GPRC5A shRNA-treated TREx-BCBL1-Rta cells. In (**A**,**B**,**D**,**E**), data are presented as mean ± SD. * *p* < 0.05, ** *p* < 0.01, *** *p* < 0.001. All repeats are biological (n = 3).

Given that expression of GPRC5A is strongly induced by RTA during KSHV lytic reactivation, and its transcript is additionally stabilised by m^6^A modification, we hypothesised that GPRCA5 may be important for KSHV lytic replication. To investigate this hypothesis, GPRC5A was depleted from TREX-BCBL1-Rta cells by lentiviral transduction, expressing two alternative GPCR5A-targeting shRNAs and knockdown, confirmed by RT-qPCR (Figure 4A). During latency, 40% and 60% decreases in *GPRC5A* mRNA levels were achieved relative to cells treated with a scrambled control shRNA, whereas both shRNAs inhibited the upregulation of *GPRC5A* mRNA during reactivation, leading to a more significant reduction of 80% during lytic replication. Upon reactivation of control and GPRC5A knockdown TREx-BCBL1-Rta cells, a slight but significant 18% reduction in the levels of the early lytic *ORF57* mRNA with a greater 29–43% decrease in the late lytic *ORF47* transcript was observed, suggesting cumulative inhibition of the KSHV lytic cycle (Figure 4B). Similarly, reduced ORF57 (early lytic) and ORF65 (late lytic) protein expression was identified in GPRC5A-depleted cells (Figure 4C,D). Furthermore, reinfection assays to measure infectious virion production were performed using supernatant harvested from scrambled and GPRC5A-depleted TREx-BCBL1-Rta cells to reinfect naïve HEK 293T cells (Figure 4E). Measuring *ORF57* mRNA levels in these reinfected HEK 293T cells indicated that knockdown of GPRC5A lead to a significant 38–56% decline in the production of infectious virus particles relative to cells treated with a scrambled shRNA. Together, these experiments suggest that GPRC5A is upregulated during KSHV reactivation to enhance lytic replication and efficient virion production.

### 3.5. GPRC5A Inhibits NFκB Signalling to Support KSHV Lytic Replication

**Figure 5:**
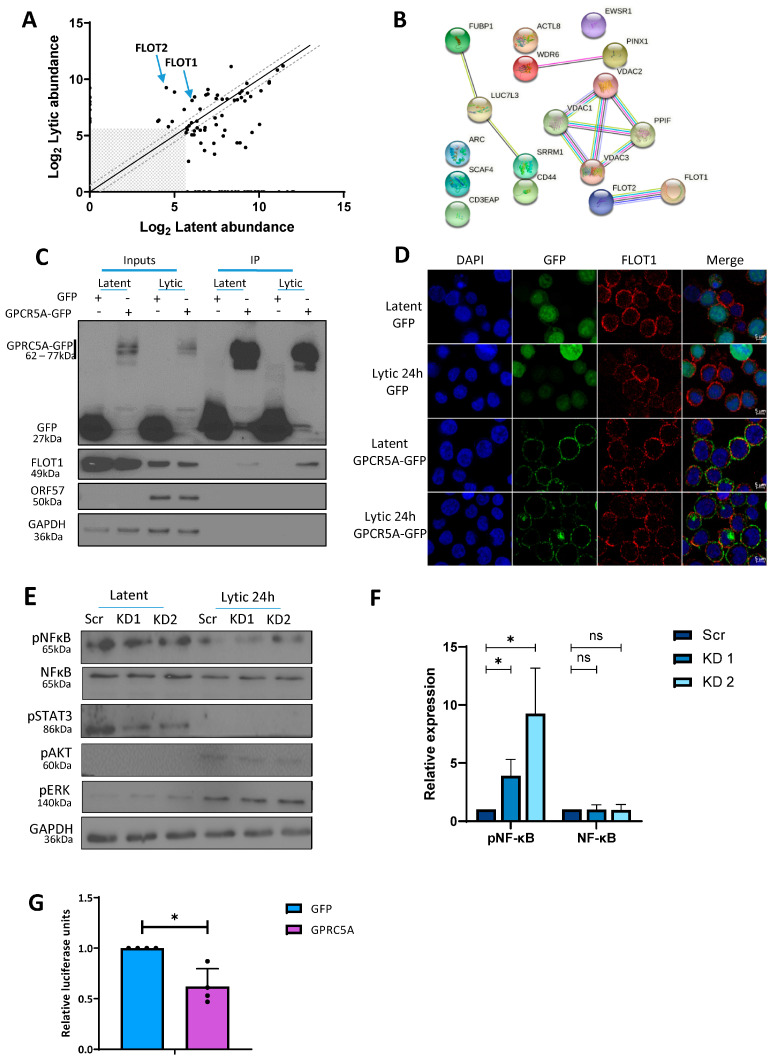
GPRC5A interacts with members of the Flotillin family and affects NFκB signalling. (**A**) Proteins with >1.5 enrichment in FLAG immunoprecipitates from GPRC5A-FLAG-overexpressing TREx-BCBL1-Rta cells compared with parental TREx-BCBL1-Rta cells after TMT mass spectrometry analysis (grey box: data points with less than 50 abundance in both latent and lytic cells were excluded). (**B**) STRING interaction map showing proteins with >1.4-fold interaction with GPRC5A in lytic replication compared with latency in TREx-BCBL1-Rta cells after TMT mass spectrometry analysis. (**C**) Immunoprecipitation of GFP or GPRC5A-GFP from transduced latent and lytic TREx-BCBL1-Rta cells showing increased co-immunoprecipitation of FLOT1 in lytic replication. (**D**) Immunofluorescence analysis of FLOT1 localisation in latent and lytic TREx-BCBL1-Rta cells transduced with a GFP control (upper two) or GFP-tagged (lower two) GPRC5A-expressing plasmid. (**E**) Representative Western blots of NFκB, pNFκB, pSTAT, pAKT, pERK and GAPDH protein expression in latent and lytic GPRC5A-depleted cells. (**F**) Densitometry quantification of NFκB and pNFκB immunoblots was performed using ImageJ software, and is shown as a percentage relative to the loading control, GAPDH. (**G**) Luciferase reporter assay from HEK 293Ts co-transfected with GFP or GPRC5A-GFP alongside various described signalling reporters with transcription factor binding sites attached to a luciferase reporter plasmid. Data presented are relative to an internal firefly control. In (**F**,**G**), data are presented as mean ± SD. * *p* < 0.01. All repeats are biological (n = 3).

Multiple studies have reported a role for membrane-bound G-protein-coupled receptors (GPCRs) at the top of signalling cascades [60]. Interestingly, KSHV encodes a constitutively active GPCR (vGPCR) during lytic replication, which participates in oncogenic signalling through the ERK1/MAPK pathway to induce angiogenesis. To investigate why KSHV also upregulated host cell GPRC5A during lytic replication, we firstly mapped its viral and cellular cofactors. To identify such interactors, TREx-BCBL1-Rta cells were transduced with a constitutive expression GPRC5A-FLAG construct, and global quantitative proteomic analysis was performed in duplicate on FLAG immunoprecipitates from latent and reactivated TREx BCBL1-Rta cell lysates (Figure 5A). We identified 16 proteins with greater than 1.5-fold interaction with GPRC5A during lytic replication in both replicates (Table S3). Among these proteins, both members of the flotillin family, FLOT1 and FLOT2, were present at 4-fold and 27-fold higher levels in the lytic phase, respectively (Figure 5B). This result was confirmed by coimmunoprecipitation of FLOT1 in GPRC5A-GFP immunoprecipitates from latent and lytic TREx BCBL1-Rta cells, where increased FLOT1 protein was detected in association with GPRC5A during KSHV lytic replication (Figure 5C). Furthermore, immunofluorescence studies revealed that FLOT1 and GPRC5A-GFP were both concentrated at the cell periphery in membrane-associated microdomains reminiscent of lipid rafts in both replication states, with enhanced co-localisation observed during lytic replication (Figure 5D). Previous studies have suggested that flotillins modulate the activity of several membrane proteins by enhancing their surface expression [61]. As GPCRs are commonly associated with cellular signalling pathways, we hypothesise that improved organisation of GPRC5A into these membrane-associated microdomains during lytic replication may allow for increased signal transduction through this protein [62].

Given the widespread role of GPCRs in cellular signalling pathways, many of which are of central importance within KSHV lytic replication, we sought to identify whether upregulation of GPRC5A affects downstream signalling cascades, which may impact KSHV lytic replication. Through Western blot analysis of key signalling proteins, comparing scrambled control and GPRC5A-depleted TREx-BCBL1-Rta cells undergoing lytic replication, we observed little to no change in pERK, pAKT or pSTAT3 levels. However, the results identified a significant increase in levels of p-NFĸB S536 (p65) during lytic replication in cells depleted for GPRC5A compared to those treated with a scrambled control, indicating an increase in NFĸB signalling (Figure 5E,F). This suggests that lytically expressed GPRC5A represses NFĸB signalling, which aligns with previous results suggesting that NFκB is a negative regulator of numerous lytic-cycle-associated genes [63]. To support this observation and determine whether GPRC5A directly regulates NFκB signalling, we transfected HEK 293T cells with GPRC5A-GFP and GFP control expression constructs alongside an NFκB-responsive luciferase reporter plasmid (Figure 5G). Analysis showed that luciferase activity was significantly attenuated in HEK 293T cells transfected with GPRC5A-GFP compared to those transfected with control GFP, suggesting GPCR5A-mediated inhibition of NFĸB activity. Taken together, these results are consistent with previous observations showing that NFĸB is reduced during KSHV lytic replication due to its repressive properties [64], and provide evidence that GPRC5A contributes to this downregulation.

## 4. Discussion

**Figure 6:**
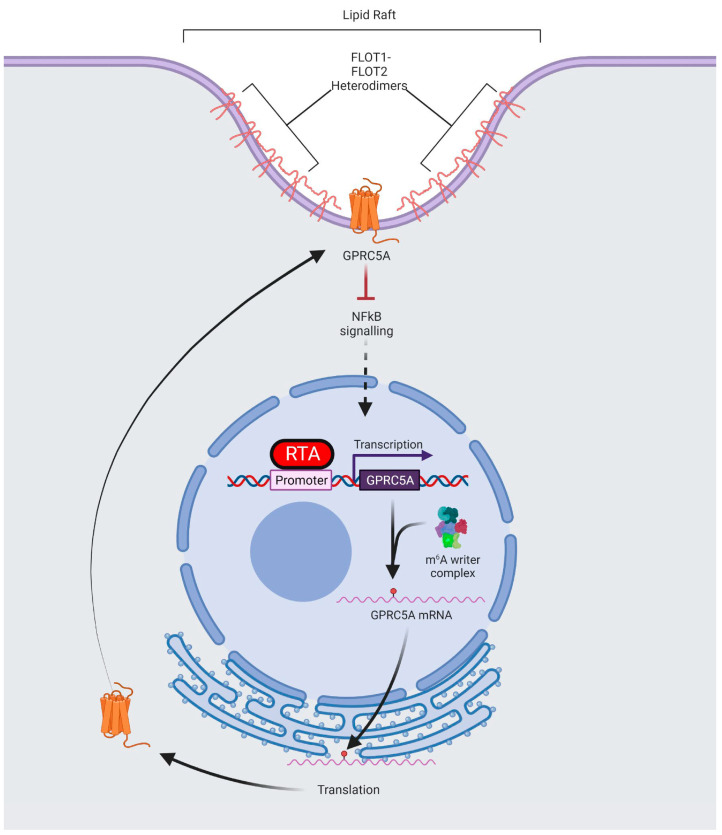
Schematic showing potential regulation of GPRC5A gene expression during KSHV lytic replication. At the onset of KSHV lytic replication, the viral lytic master regulator RTA transactivates the GPRC5A promoter, increasing transcription of GPRC5A transcripts, which then become m^6^A-modified by the m^6^A methyltransferase complex. Methylation of GPRC5A mRNA increases its stability in cis, allowing translation of a larger pool of GPRC5A transcripts, and therefore, enhanced protein expression. Once translated, GPRC5A is transported to the cell membrane, where it is organised into lipid rafts composed of members of the flotillin family. As a membrane-bound GPCR, GPRC5A inhibits NFκB to enhance KSHV lytic replication.

Although m^6^A is known to play a crucial regulatory role in the life cycle of numerous viruses, most studies to date have investigated the contribution of the modification to viral transcripts, rather than cellular gene expression. Here, we show that a number of host cell transcripts with differential m^6^A modification during the latent and lytic replication phases correspond to their increased expression during lytic replication. In addition, focussing on one of these differentially m^6^A-modified transcripts, we show that GPCR5A enhances KSHV lytic replication by acting as a novel inhibitor of NFκB signalling (Figure 6).

Given the fundamental role m^6^A plays in directing RNA fate and dynamics, targeting of the modification provides an opportunity to develop therapeutics that modulate the expression of genes essential for disease pathogenesis. During KSHV lytic reactivation, most cellular transcripts are degraded by a program of host cell shutoff orchestrated by the viral endonuclease protein, SOX [51,52]. Therefore, genes that are resistant to the global pattern of downregulation are more likely to be biologically relevant for KSHV lytic replication. As a result, we concentrated on m^6^A-modified transcripts with increased abundance in the lytic life cycle.

We identified four transcripts, *GPRC5A*, *STC1*, *FOSB* and *ZFP36L1*, with increased mRNA levels during lytic replication; however, this effect could be modulated through the depletion of m^6^A machinery components, suggesting that m^6^A modification of these mRNAs contributes to their abundance. Accordingly, mutation of m^6^A sites within the *GPRC5A* transcript demonstrates that methylation increases its stability. Although m^6^A is generally thought to destabilise A/U base pairing, several studies have demonstrated that in certain sequence/structure-dependent contexts, m^6^A improves mRNA stability, highlighting the importance of m^6^A examination on a site-specific basis [18,22,65]. Thus, we hypothesise that m^6^A allows increases in GPRC5A protein expression through tuneable improvements in mRNA stability, increasing the pool of mRNAs available for translation at any one time.

The importance of the m^6^A-modified *GPRC5A* transcript in KSHV lytic replication is further reinforced upon depletion studies, which show reduced lytic protein production and infectious virion production. Interestingly, GPCR5A was directly upregulated by ectopic expression of RTA, the master regulator of KSHV lytic replication. RTA binds the promoters of cellular and viral genes using its N-terminal DNA binding domain, while its C-terminal transactivation domain co-opts several host transcription factors to activate transcription [8,10,12]. Analysis of previous published RTA-CHIP-seq datasets revealed RTA-binding sites in the GPRC5A promoter, reinforcing our finding that RTA upregulates these genes to play an important function within KSHV lytic replication [59]. Interestingly, GPRC5A was identified as one of many RTA-inducible cellular plasma membrane proteins that serve as key regulators of signalling and facilitate transformation of the host cell environment to support lytic replication [59].

As GPRC5A appears to enhance lytic replication, we hypothesise that identification of interaction partners might provide an important insight into the role of GPCR5A within KSHV lytic replication. Quantitative mass spectrometry revealed that prominent interactors of GPRC5A were common constituents of plasma membranes, including the lipid raft structural proteins, namely, members of the flotillin family, FLOT1 and FLOT2. We hypothesise that this interaction signifies the organisation of GPRC5A, as with other GPCRs, into lipid rafts at the plasma membrane. Our results suggest that the increased interaction of flotillins with GPRC5A during lytic replication allows greater packaging of GPRC5A into lipid rafts to enhance its surface expression and provide a microenvironment conducive for signal transduction [66].

Previous research has identified that GPRC5A is a membrane protein, whose ligand is yet to be discovered, capable of activating cellular signalling pathways, including STAT3 and EGFR [67,68,69,70]. Although we tested whether GPRC5A was capable of modulating these pathways during KSHV reactivation by comparing scrambled control and GPRC5A-depleted TREx-BCBL1-Rta cells, we were unable to detect EGFR activity in either latent or lytic replication states. Furthermore, STAT3 signalling, although rapidly inhibited during lytic replication, was unaffected by the depletion of GPRC5A. Interestingly, however, when screening other key regulators of signalling, we identified an increase in levels of pNFĸB S536 during the lytic replication phase in cells depleted for GPRC5A compared to the scrambled control, indicating an increase in NFĸB signalling upon GPRC5A knockdown. This was further corroborated by overexpression studies showing GPRC5A-mediated repression of an NFκB-responsive luciferase reporter plasmid. The activation of NF-κB is central to KSHV infection, by modulating viral gene expression, and for the pathogenesis of KSHV-associated malignancies, via the induction of inflammatory mediators and the expression of antiapoptotic genes. NFκB is strongly activated during KSHV latency by the virally encoded latency factor vFLIP, enhancing the expression of latent transcripts while inhibiting lytic-cycle-associated genes [63,64]. In contrast, NFκB is a negative regulator of numerous lytic-cycle-associated genes, due to NFκB competing with RTA for the binding of the Notch transcription factor RBP-JK and preventing the transcription of RTA-responsive genes [64,71]. Our results, therefore, highlight a novel role of GPRC5A in regulating NFκB signalling during the KSHV lytic replication cycle.

In summary, we show that m^6^A regulates the fate and function of cellular transcripts important for KSHV lytic replication. We identified the RTA-inducible gene GPRC5A, whose mRNA and protein abundance can be fine-tuned by m^6^A-dependent alterations in RNA stability. Finally, we demonstrate that GPRC5A is important in moderating the inhibitory effect of NFκB signalling on KSHV lytic replication. Our work contributes further evidence to the fundamental nature of m^6^A in the post-transcriptional regulation of gene expression within viral infections. Given the ubiquitous distribution of RNA modifications across RNAs critical to viral infection, it seems likely that epitranscriptomics will emerge as a key determinant in the outcome of viral infections and a major contributor to the development of antiviral therapeutics.

## Data Availability

The mass spectrometry proteomics data have been deposited into the ProteomeXchange Consortium via the PRIDE partner repository. TREx-BCBL1-RTA GPRC5A-FLAG Immunoprecipitation LC-MS. Project accession: PXD039669. Data and constructs will be available from the corresponding author upon request.

## Supplementary Materials

The following supporting information can be downloaded at https://www.mdpi.com/article/10.3390/v15061381/s1. The following supplementary information is included. Figure S1. Differential m^6^A modification of cellular transcripts during KSHV lytic replication. Figure S2. Ingenuity pathway analysis (IPA). Figure S3. Mutation of DRACH sequences within GPRC5A to abolish the differentially modified m^6^A peak during KSHV lytic replication. Figure S4. RTA binds the promoters internally within *GPRC5A* and *ZFP36L1*. Table S1. List of primer sequences. Table S2. List of transcripts with differentially modified m^6^A peaks during KSHV lytic replication. Table S3. List of host cell proteins with enhanced interaction of GPCR5A during KSHV lytic replication.
