# Supplementary material for: m6A Regulates the Stability of Cellular Transcripts Required for Efficient KSHV Lytic Replication
Source: Viruses. 2023 Jun 16;15(6):1381. doi: 10.3390/v15061381 (PMC10303434; doi:10.3390/v15061381)
Supplement: Supplementary file 1 [file viruses-15-01381-s001.zip › viruses-2369878-supplementary.pdf]

m^6A regulates the stability of cellular transcripts required for efficient KSHV lytic replication

Oliver Manners, Belinda Baquero-Perez, Timothy J. Mottram, Ivaylo D. Yonchev, Christopher J. Trevelyan, Katherine L. Harper, Sarah Menezes, Molly R. Patterson, Andrew Macdonald, Stuart A. Wilson, Julie L. Aspden & Adrian Whitehouse

Supporting Information

Supplementary Figures S1-S4
Supplementary Tables S1-S3

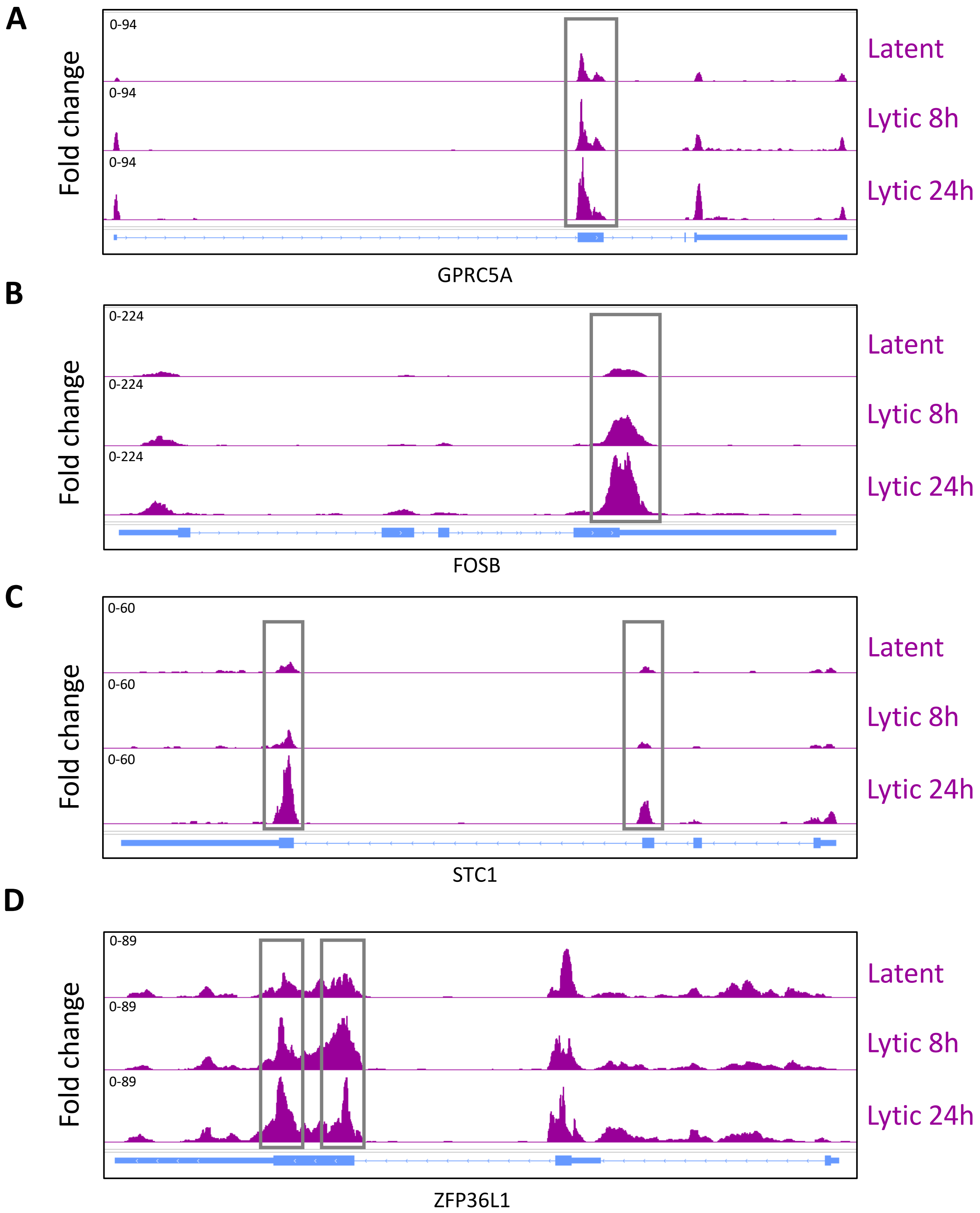
A
Fold change
0-94
0-94
0-94
Latent
Lytic 8h
Lytic 24h
GPRC5A
B
Fold change
0-224
0-224
0-224
Latent
Lytic 8h
Lytic 24h
FOSB
C
Fold change
0-60
0-60
0-60
Latent
Lytic 8h
Lytic 24h
STC1
D
Fold change
0-89
0-89
0-89
Latent
Lytic 8h
Lytic 24h
ZFP36L1

E

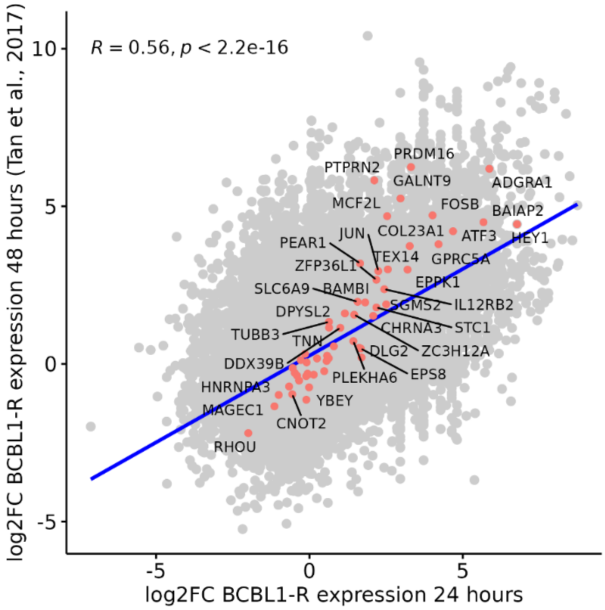
R = 0.56, p < 2.2e-16
log2FC BCBL1-R expression 48 hours (Tan et al., 2017)
log2FC BCBL1-R expression 24 hours
10
5
0
-5
-5
0
5
PRDM16
PTPRN2
GALNT9
ADGRA1
MCF2L
FOSB
BAIAP2
COL23A1
JUN
ATF3
HEY1
PEAR1
TEX14
GPRC5A
ZFP36L1
EPPK1
SLC6A9
BAMBI
SGMS2
IL12RB2
DPYSL2
CHRNA3
STC1
TUBB3
TNN
DLG2
ZC3H12A
DDX39B
PLEKHA6
EPS8
HNRNPA3
YBEY
MAGEC1
CNOT2
RHOU

F (i) **(ii)**

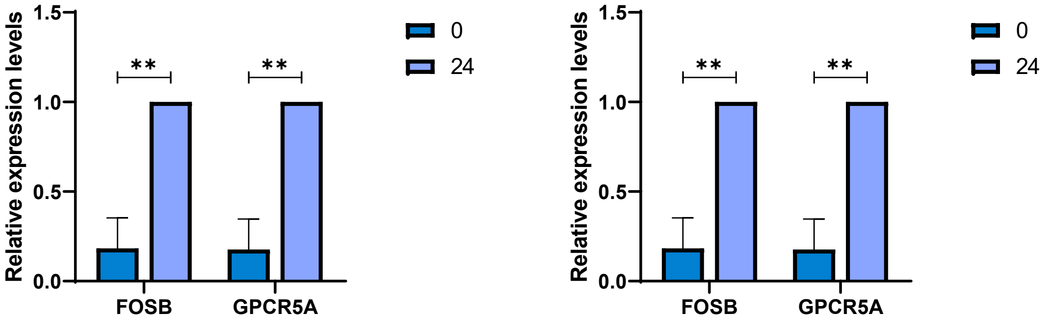
Relative expression levels
1.5
1.0
0.5
0.0
**
**
FOSB
GPCR5A
0
24
Relative expression levels
1.5
1.0
0.5
0.0
**
**
FOSB
GPCR5A
0
24

G

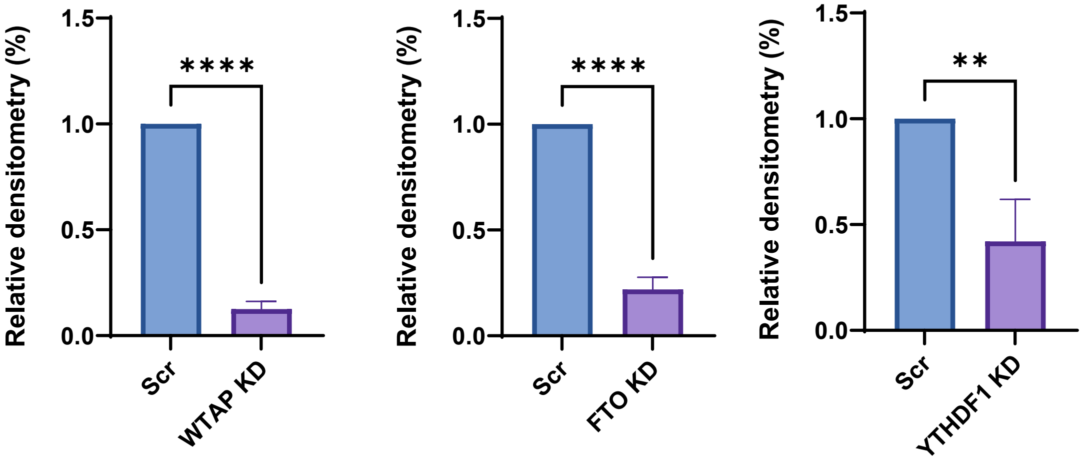
Relative densitometry (%)
1.5
1.0
0.5
0.0
****
Scr
WTAP KD
Relative densitometry (%)
1.5
1.0
0.5
0.0
****
Scr
FTO KD
Relative densitometry (%)
1.5
1.0
0.5
0.0
**
Scr
YTHDF1 KD

H

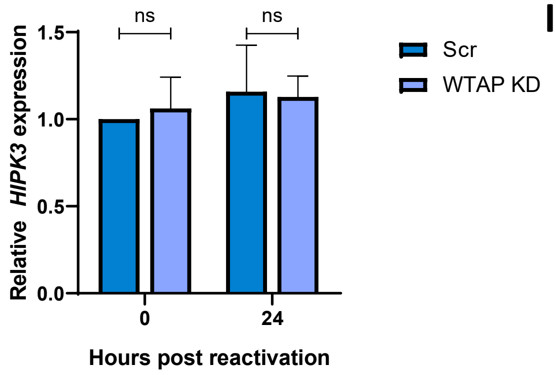

I

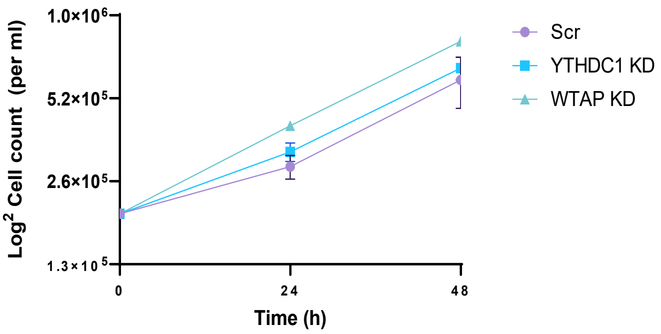

Figure S1. Differential m^{6}A modification of cellular transcripts during KSHV lytic replication. Genome sequencing tracks for (A) GPRC5A, (B) FOSB, (C) STC1 and (D) ZFP36L1 from latent and lytic TREX cells depicting input (dark blue) and m^{6}A IP (purple) reads on cellular mRNAs. Differentially modified peaks showing an increase in m^{6}A modification during lytic replication are indicated by black boxes. (E) Correlation plot between the DESeq2 calculated log2-fold changes in gene expression within this study and published BCBL1-R KSHV data [49]. R marks the Pearson correlation coefficient between the datasets. Selected transcripts observed to show differential m6A peak modifications are highlighted in red. (F) RT-qPCR analysis of cellular m^{6}A-modified transcripts in (i) BCBL1 and (ii) HEK 293 rKSHV.219 cells induced for 24 hours compared to latent levels. (G) Densitometry analysis of westerns blots from Figure 1D,G,J (n=3), ****P<0.0001, **P<0.01. (H). RNA levels of HIPK3 in Scr- or WTAP shRNA-treated latent and induced TREX-BCBL-1 cells. Data presented as mean ± SD (n=3), **P<0.01. (I) Proliferation rate of Scr-, WTAP and YTHDF1 shRNA-treated cell lines. Cells were counted over 48 hours to measure cell proliferation (n=2 biologically independent samples).

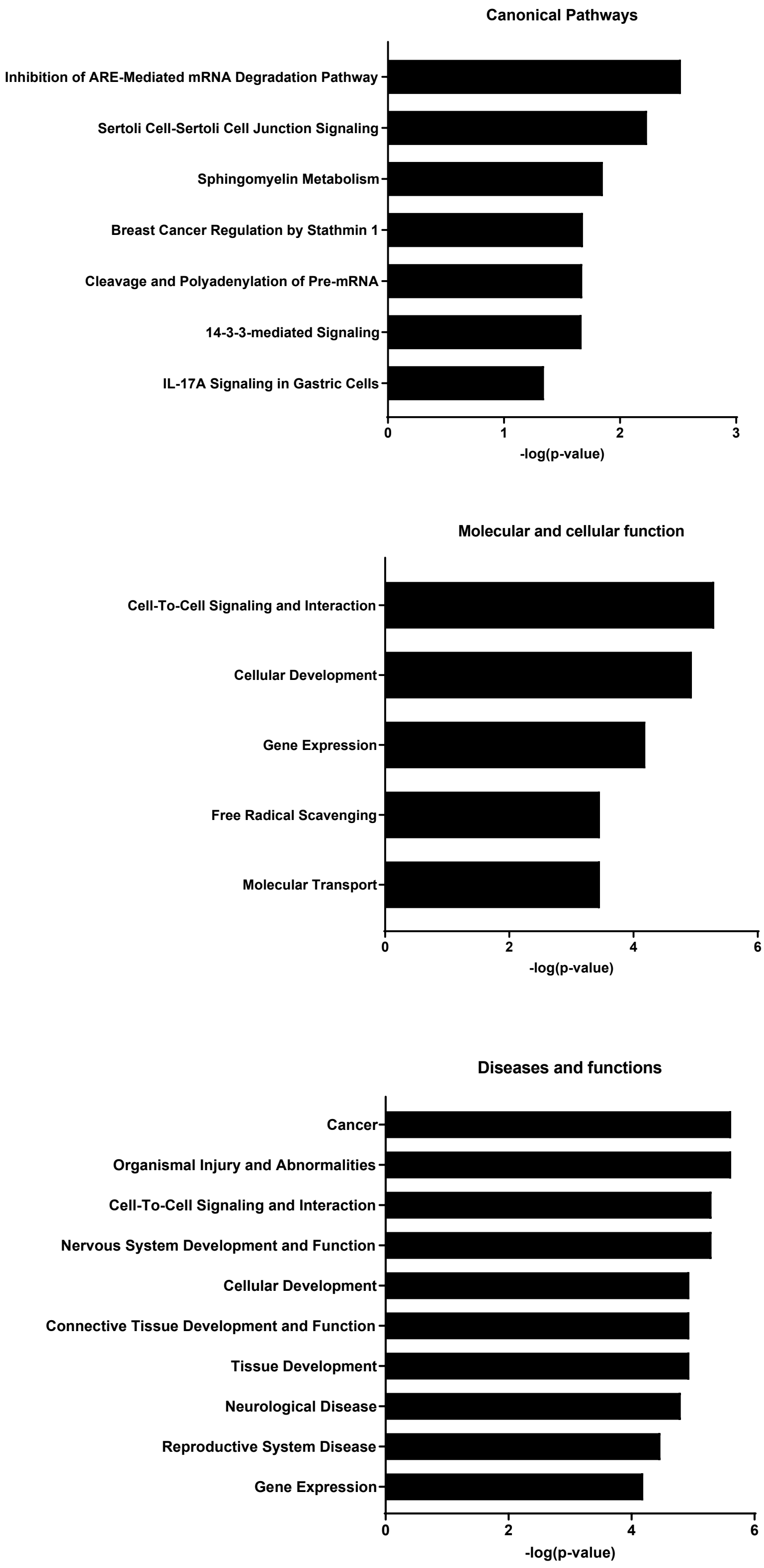

Figure S2. Ingenuity pathway analysis (IPA). Analysis identified that the differentially m^{6}A modified mRNAs were associated with pathways involved RNA processing and cell signalling. Similarly, predicted molecular and cellular functions and diseases identified cell-to-cell signalling, gene expression and cancer-related pathways.

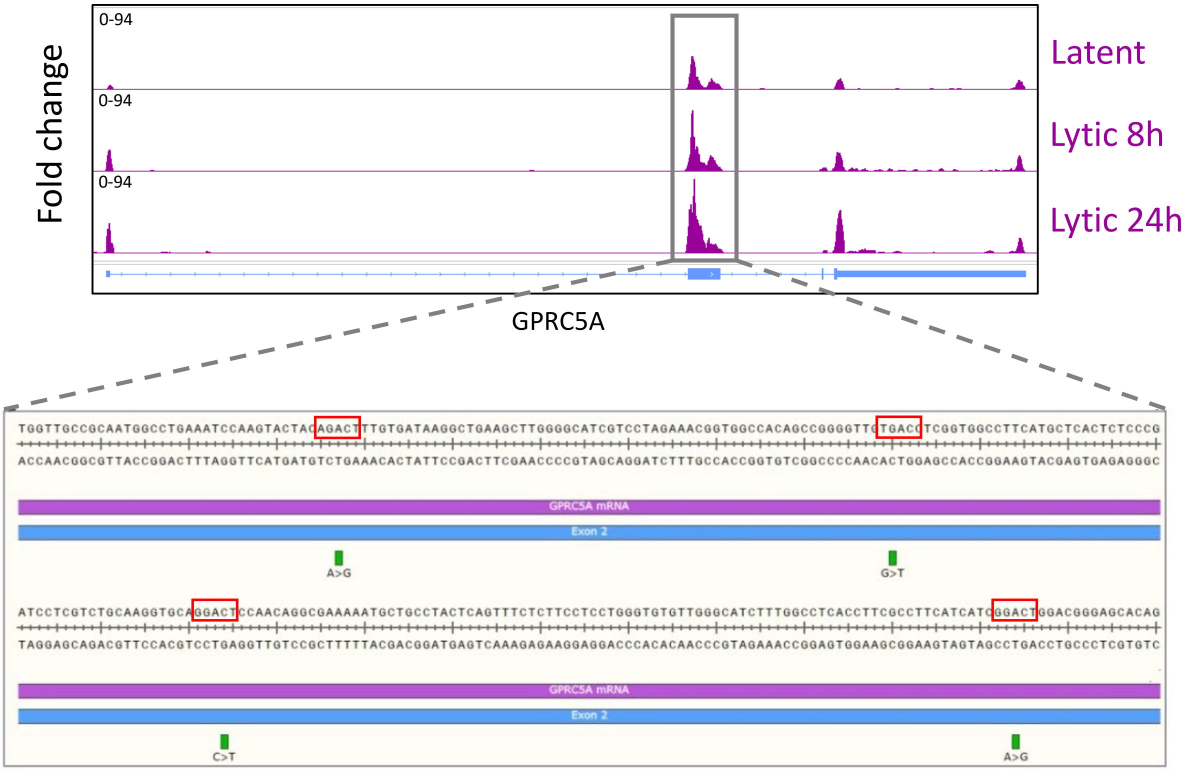

Figure S3. Mutation of DRACH sequences within GPRC5A to abolish the differentially modified m^{6}A peak during KSHV lytic replication. Site directed mutagenesis was carried out to abrogate 4 DRACH sequences of GPRC5A in close proximity to the m^{6}A peak identified as differentially modified during KSHV lytic replication. Consensus DRACH sequences are highlighted with red box.

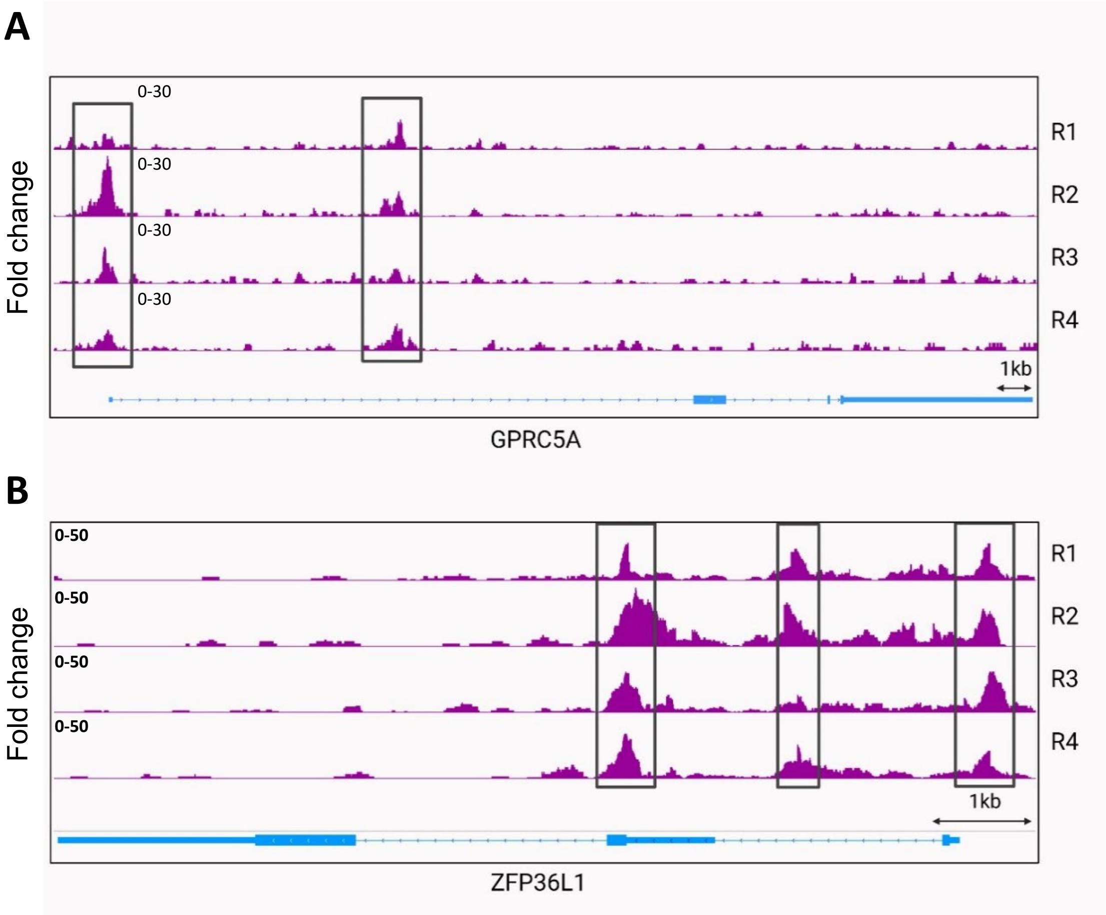

Figure S4. RTA binds the promoters and internally within *GPRC5A* and *ZFP36L1*. Genome sequencing tracks from lytic TREX cells depicting RTA-CHIP peaks within (A) *GPRC5A* and (B) *ZFP36L1* genes. Experiments were performed in Papp et al 2019 (52).

Gene name	Forward	Reverse
FOSB	5'-GAAATGCCCGGTTCCTTC-3'	5'-GAGGGTGGGTTGCACAAG-3'
FTO	5'-TCTGACCCCCAAAGATGATG-3'	5'-CTCGGAGAATTAGTTTAGGATATTTCA-3'
GAPDH	5'-TGTCAGTGGTGGACCTGAC-3'	5'-GTGGTCGTTGAGGGCAATG-3'
GPRC5A	5'-CCTTTCCCTGTTGGTGATTCT -3'	5'-AGACATTGACGTTGGTCCTATTC-3'
JUN	5'-CGCCTGATAATCCAGTCCA-3'	5'-TTCTTGGGGCACAGGAACT-3'
ORF47	5'-CGCGGTCGTTCGAAGATTG-3'	5'-CGAGTCTGACTTCCGCTAA-3'
ORF57	5'-GCCATAATCAAGCGTACTGG-3'	5'-GCAGACAAATATTGCGGTGT-3'
PLEKHA6	5'-CCACCAGGCAAGAGGTAGAG-3'	5'-GCACAACGCCAACTTTGTT-3'
STC1	5'-AGGCGGAGCAGAATGACTC-3'	5'-GTTGAGGCAACGAACCACTT-3'
WTAP	5'-TTCCCAAGAAGGTTCGATTG-3'	5'-TGCAGACTCCTGCTGTTGTT-3'
YTHDF1	5'-ATAACCAGCTCCGGCACAT-3'	5'-GGGAGTTTGTGACCGGTTT-3'
ZC3H12A	5'-TCTGTGGGAATTTGAGGACAG-3'	5'-GTGGATCTCCGTGGATGAATAG-3'
ZFP36L1	5'-GCGAAGTTTTATGCAAGGGTAA-3'	5'-GTGCCCACTGCCTTTCTG-3'
FOSB Control	5'-GGAGAGCTGGTGACTTTGGG-3'	5'-AGAGCCAACAGTCAGCTGGG-3'
FOSB m^6A	5'-CTCCCTCCTCGCTCTGTGAA-3'	5'-CAAGTCTCTCTCCCCCATGT-3'
GAPDH 1	5'-GCATCTTCTTTTGCGTCGCC-3'	5'-TTGACTCCGACCTTCACCTTCC-3'
GAPDH 2	5'-TGCACCACCAACTGCTTA-3'	5'-ATGAGTCCTTCCACGATACC-3'
GPRC5A control	5'-CCGAGATCTAATCTCCCCCTA-3'	5'-GGGCTTGTGCTAGTGAGGTC-3'
GPRC5A m^6A	5'-CTCACTCTCCCGATCCTCGT-3'	5'-GAAACTGAGTAGGCAGCATT-3'
JUN Ctrl	5'-AGCGCCTGATAATCCAGTCC-3'	5'-ATCTGTCACGTTCTTGGGGC-3'
JUN m^6A	5'-ACCTTGAAAGCTCAGAACTCGG-3'	5'-TAAGCTGTGCCACCTGTTCC-3'
SLC39A14 control	5'-GCAGGATCTAATACATCGGTATGG-3'	5'-TGGTTGAGTAGGGCCTTCAG-3'
SLC39A14 m^6A	5'-GGACAGATCCAGATTGGGTAG-3'	5'-AGGGCCCGACTTCCAGT-3'
ZFP36L1 control	5'-GGCACACACACATTAAGATGAA-3'	5'-AGAGAAATAGAAAGCGACGGT-3'
ZFP36L1 m^6A site 1	5'-GGTTGCCTGCTGGACAGAAA -3'	5'-TTCTGGTGGAACTTGGAGCTG-3'
ZFP36L1 m^6A site 2	5'-CAGGATTCTCTCTCGGACCA -3'	5'-TCCAAGGTCGGGGAGTCT-3'
WTAP shRNA KD	TRCN0000231423	
FTO shRNA KD	TRCN0000246247	
YTHDF1 shRNA KD	TRCN0000286871	
GPRC5A shRNA KD1	TRCN0000005628	
GPRC5A shRNA KD2	TRCN0000005632	

Table S1. List of primer sequences.

Dark grey = low expression

	Raw values (IGV)						Relative values (IGV)						Relative expression change (DESeq2)			
	R1			R2			R1			R2			R1+R2			
Gene	0h	8h	20h	0h	8h	20h	0h	8h	20h	0h	8h	20h	0h	20h	Methlyation change	Expression change
ADIRF	0.16	0.08	0.05	1.67	0.67	0.09	1.00	0.50	0.31	1.00	0.40	0.05			Decrease	
ADGRA1	0.04	0.08	0.06	0.05	0.16	0.16	1.00	2.00	1.50	1.00	3.20	3.20			Increase	
ATF3	0.04	0.04	0.32	0.05	0.10	0.19	1.00	1.00	8.00	1.00	2.00	3.80	1.00	25.67	Increase	Increase
BAIAP2	0.02	0.03	0.17	0.12	0.19	0.21	1.00	1.50	8.50	1.00	1.58	1.75	1.00	51.12	Increase	Increase
BAMBI	1.39	1.04	1.58	5.76	7.04	1.49	1.00	0.75	1.14	1.00	1.22	0.26	1.00	3.53	Decrease	Increase
BRD9	1.02	0.82	0.89	16.00	10.00	2.14	1.00	0.80	0.87	1.00	0.63	0.13	1.00	0.94	Decrease	Decrease
CHRNA3	0.02	0.04	0.38	0.04	0.25	0.29	1.00	2.00	19.00	1.00	6.25	7.25	1.00	4.23	Increase	Increase
CNOT2	0.24	0.21	0.21	2.76	2.30	0.24	1.00	0.88	0.88	1.00	0.83	0.09	1.00	0.68	Decrease	Decrease
COL23A1	0.08	0.12	0.05	0.30	0.24	0.09	1.00	1.50	0.63	1.00	0.80	0.30	1.00	9.64	Decrease	Increase
DLG2	0.01	0.01	0.05	0.05	0.04	0.02	1.00	1.00	5.00	1.00	0.87	0.43	1.00	3.07	Decrease	Increase
DPYSL2	0.05	0.02	0.34	0.03	0.31	0.28	1.00	0.40	6.80	1.00	10.33	9.33	1.00	2.23	Increase	Increase
EPPK1	0.33	0.23	0.23	1.93	1.87	0.72	1.00	0.70	0.70	1.00	0.97	0.37	1.00	9.18	Decrease	Increase
EPS8	0.05	0.04	0.11	0.07	0.20	0.20	1.00	0.80	2.20	1.00	2.86	2.86	1.00	3.18	Increase	Increase
FOSB	0.06	0.12	1.74	0.63	3.02	1.94	1.00	2.00	29.00	1.00	4.79	3.08	1.00	16.15	Increase	Increase
GALNT9	0.01	0.04	0.04	0.05	0.06	0.06	1.00	4.00	4.00	1.00	1.20	1.20	1.00	7.83	Increase	Increase
GOLM1	0.22	0.20	0.09	1.06	1.91	0.09	1.00	0.91	0.41	1.00	1.80	0.08	1.00	1.47	Decrease	Increase
GPRC5A	0.07	0.07	0.70	0.88	2.79	1.18	1.00	1.00	10.00	1.00	3.17	1.34	1.00	18.49	Increase	Increase
HEY1	0.01	0.03	0.22	0.03	0.80	0.22	1.00	3.00	22.00	1.00	26.67	7.33	1.00	109.04	Increase	Increase
hnRNPA3	0.31	0.21	0.04	3.07	3.31	0.15	1.00	0.68	0.13	1.00	1.08	0.05	1.00	0.50	Decrease	Decrease
IL12RB2	0.01	0.01	0.36	0.04	0.08	0.06	1.00	1.00	36.00	1.00	2.00	1.50	1.00	5.43	Increase	Increase
ILF3	0.04	0.07	0.47	0.10	2.08	0.73	1.00	1.75	11.75	1.00	20.80	7.30	1.00	0.93	Decrease	Decrease
JTB	0.09	0.05	0.02	1.07	0.16	0.04	1.00	0.56	0.22	1.00	0.15	0.04	1.00	0.96	Decrease	Decrease
JUN	0.45	0.39	6.46	5.41	19.00	3.64	1.00	0.87	14.36	1.00	3.51	0.67	1.00	4.74	Increase	Increase
MAGEC1	0.10	0.10	0.05	1.14	0.65	0.07	1.00	1.00	0.50	1.00	0.57	0.06	1.00	0.45	Decrease	Decrease
MCF2L	0.01	0.07	0.12	0.37	0.44	0.35	1.00	7.00	12.00	1.00	1.19	0.95	1.00	5.79	Increase	Increase
PABPN1	0.47	0.33	0.44	1.85	1.52	0.17	1.00	0.70	0.94	1.00	0.82	0.09	1.00	0.68	Decrease	Decrease
PCF11	0.44	0.28	0.37	2.74	2.77	0.38	1.00	0.64	0.84	1.00	1.01	0.14	1.00	1.49	Decrease	Increase
PCNX1	0.04	0.04	0.60	0.62	1.16	1.16	1.00	1.00	15.00	1.00	1.87	1.87	1.00	0.82	Increase	Decrease
PEAR1	0.01	0.03	0.06	0.08	0.32	0.17	1.00	3.00	6.00	1.00	4.00	2.13	1.00	3.14	Increase	Increase
PILRB	0.06	0.04	0.48	0.16	1.03	0.63	1.00	0.67	8.00	1.00	6.44	3.94	1.00	1.73	Increase	Increase
PLEKHA6	0.02	0.05	0.30	0.05	0.38	0.47	1.00	2.50	15.00	1.00	7.60	9.40	1.00	2.70	Increase	Increase
PPP2R2D	1.01	0.58	0.77	5.18	7.96	1.18	1.00	0.57	0.76	1.00	1.54	0.23	1.00	0.90	Decrease	Decrease
PRDM16	0.03	0.05	0.11	0.01	0.17	0.22	1.00	1.67	3.67	1.00	17.00	22.00	1.00	9.91	Increase	Increase
PTPRN2	0.01	0.04	0.10	0.03	0.48	0.59	1.00	4.00	10.00	1.00	16.00	19.67	1.00	4.33	Increase	Increase
RBM12B	4.13	3.07	0.90	58.00	32.00	4.64	1.00	0.74	0.22	1.00	0.55	0.08	1.00	1.10	Decrease	Increase
RFTN1	0.30	0.27	0.02	1.68	0.99	0.12	1.00	0.90	0.07	1.00	0.59	0.07	1.00	0.63	Decrease	Decrease
RhoU	0.24	0.19	0.92	2.08	4.37	3.81	1.00	0.79	3.83	1.00	2.10	1.83	1.00	0.25	Increase	Decrease
SDF4	0.02	0.06	0.33	0.11	0.50	0.42	1.00	3.00	16.50	1.00	4.55	3.82	1.00	1.55	Increase	Increase
SGMS2	0.06	0.11	0.89	0.37	5.77	1.13	1.00	1.83	14.83	1.00	15.59	3.05	1.00	5.67	Increase	Increase
SLC25A25	0.06	0.05	0.26	0.09	1.21	0.12	1.00	0.83	4.33	1.00	13.44	1.33	1.00	3.25	Increase	Increase
SLC6A9	0.03	0.05	0.32	0.08	1.45	0.27	1.00	1.67	10.67	1.00	18.13	3.38	1.00	2.97	Increase	Increase
STC1	0.03	0.09	0.52	0.21	0.40	0.45	1.00	3.00	17.33	1.00	1.90	2.14	1.00	4.54	Increase	Increase
TEX14	0.01	0.03	0.09	0.06	0.03	0.13	1.00	3.00	9.00	1.00	0.50	2.17	1.00	5.88	Increase	Increase
TIFA	0.76	0.38	0.09	5.91	3.84	0.40	1.00	0.50	0.12	1.00	0.65	0.07	1.00	0.97	Decrease	Decrease
TNN	0.32	0.38	1.34	5.63	8.15	3.74	1.00	1.19	4.19	1.00	1.45	0.66	1.00	1.56	Decrease	Increase
TNRC6A	1.33	1.04	2.34	9.47	18.00	2.35	1.00	0.78	1.76	1.00	1.90	0.25	1.00	0.72	Decrease	Decrease
TUBB3	0.04	0.04	0.04	0.18	0.20	0.08	1.00	1.00	1.00	1.00	1.11	0.44	1.00	1.55	Decrease	Increase
UAP56	0.01	0.01	0.62	0.02	0.33	0.78	1.00	1.00	62.00	1.00	16.50	39.00	1.00	2.01	Increase	Increase
USP48	0.07	0.08	0.21	0.68	1.12	0.18	1.00	1.14	3.00	1.00	1.65	0.26	1.00	0.79	Decrease	Decrease
YBEY	0.21	0.18	0.09	2.53	1.32	0.15	1.00	0.86	0.43	1.00	0.52	0.06	1.00	0.93	Decrease	Decrease
YTHDF3	1.72	1.57	2.07	12.00	23.00	3.64	1.00	0.91	1.20	1.00	1.92	0.30	1.00	0.76	Decrease	Decrease
ZBTB21	0.03	0.14	0.31	0.13	1.17	0.59	1.00	4.67	10.33	1.00	9.00	4.54	1.00	1.19	Increase	Increase
ZC3H12A	0.24	0.08	0.13	1.06	1.19	0.25	1.00	0.33	0.54	1.00	1.12	0.24	1.00	2.75	Decrease	Increase
ZFP36L1	0.17	0.19	2.55	1.66	3.55	2.46	1.00	1.12	15.00	1.00	2.14	1.48	1.00	4.54	Increase	Increase
ZNF12	0.20	0.18	0.35	13.00	3.16	0.35	1.00	0.90	1.75	1.00	0.24	0.03	1.00	0.99	Decrease	Decrease

Table S2. List of transcripts with differentially modified m^{6}A peaks during KSHV lytic replication. Differentially modified m6A peaks were determined relative to stable m^{6}A peaks within the same transcript during KSHV lytic replication. Raw and relativized to 0h peak intensity values are listed for the differentially modified m^{6}A peak within each cellular transcript. Sites where m6A peaks were called significant by m^{6}A viewer software are highlighted in blue. The overall change in methylation is also included.

Gene names	Lytic/Latent ratio
FLOT2	26.72925764
SRRM1	6.875840979
POLR1G	4.852941176
ACTL8	4.740740741
FLOT1	4.205438066
SCAF4	3.354297694
LUC7L3	3.269565217
PINX1	2.61774744
WDR6	2.212
EWSR1	1.787741203
ARC	1.782608696
CD44	Interaction exclusive to lytic replication
FUBP1	Interaction exclusive to lytic replication
PPIF	Interaction exclusive to lytic replication
VDAC1	Interaction exclusive to lytic replication
VDAC2	Interaction exclusive to lytic replication
VDAC3	Interaction exclusive to lytic replication

Table S3. List of host cell proteins with enhanced interaction GPCR5A during KSHV lytic replication. Global quantitative proteomic analysis was performed on FLAG immunoprecipitates from latent and reactivated TREX BCBL-1 cell lysates. Proteins with an enhanced interaction with GPCR5A during lytic replication are listed.
